# Evolution of Acute Respiratory Distress Syndrome in Emergency and Critical Care: Therapeutic Management before and during the Pandemic Situation

**DOI:** 10.3390/medicina58060726

**Published:** 2022-05-28

**Authors:** Monserrat E. Granados-Bolivar, Miguel Quesada-Caballero, Nora Suleiman-Martos, José L. Romero-Béjar, Luis Albendín-García, Guillermo A. Cañadas-De la Fuente, Alberto Caballero-Vázquez

**Affiliations:** 1 Iznalloz Health Center, Granada Metropolitan District, Andalusian Health Service, Calle Virgen de la Consolación, 12, 18015 Granada, Spain; montserrat.granados.sspa@juntadeandalucia.es; 2 Albayda La Cruz Health Center, Granada Metropolitan District, Andalusian Health Service, Calle Virgen de la Consolación, 12, 18015 Granada, Spain; miguel.quesada.caballero.sspa@juntadeandalucia.es; 3 Faculty of Health Sciences, University of Granada, Campus Universitario de Ceuta, C/Cortadura del Valle SN, 51001 Ceuta, Spain; norasm@ugr.es; 4 Statistics and Operational Research Department, University of Granada, Avda. Fuentenueva S/N, 18071 Granada, Spain; 5 Casería de Montijo Health Center, Granada Metropolitan District, Andalusian Health Service, Calle Virgen de la Consolación, 12, 18015 Granada, Spain; lualbgar1979@ugr.es; 6 Faculty of Health Sciences, University of Granada, Avenida de la Ilustración, 60, 18016 Granada, Spain; gacf@ugr.es; 7 Diagnostic Lung Cancer Unit, Broncopleural Techniques and Interventional Pulmonology Departament, Hospital Universitario Virgen de las Nieves, 18014 Granada, Spain; alberto.caballero.sspa@juntadeandalucia.es

**Keywords:** COVID-19, pneumonia, pre-hospital care, respiratory distress syndrome, systematic review

## Abstract

*Background and Objectives:* Acute respiratory distress syndrome is a life-threatening lung condition that prevents enough oxygen from getting to the lungs and blood. The causes can be varied, although since the COVID-19 pandemic began there have been many cases related to this virus. The management and evolution of ARDS in emergency situations in the last 5 years was analyzed. *Materials and Methods:* A systematic review was carried out in the PubMed and Scopus databases. Using the descriptors Medical Subject Headings (MeSH), the search equation was: “Emergency health service AND acute respiratory distress syndrome”. The search was conducted in December 2021. Quantitative primary studies on the care of patients with ARDS in an emergency setting published in the last 5 years were included. *Results:* In the initial management, adherence to standard treatment with continuous positive airway pressure (CPAP) is recommended. The use of extracorporeal membrane reduces the intensity of mechanical ventilation or as rescue therapy in acute respiratory distress syndrome (ARDS). The prone position in both intubated and non-intubated patients with severe ARDS is associated with a better survival of these patients, therefore, it is very useful in these moments of pandemic crisis. Lack of resources forces triage decisions about which patients are most likely to survive to start mechanical ventilation and this reflects the realities of intensive care and emergency care in a resource-limited setting. *Conclusions:* adequate prehospital management of ARDS and in emergency situations can improve the prognosis of patients. The therapeutic options in atypical ARDS due to COVID-19 do not seem to vary substantially from conventional ARDS.

## 1. Introduction

Acute respiratory distress syndrome (ARDS) is a viral disorder characterized by high fever, dry cough, shortness of breath (dyspnea) or breathing difficulties, and atypical pneumonia with high morbidity and mortality for which there are few treatments with conclusive results. One approach to improve such outcomes in ARDS patients has been to focus on the early and accurate identification of risk factors that can be assessed prior to hospital admission in those patients at high risk for ARDS [1]. The Acute Respiratory Distress Syndrome Diagnosis includes clinical and ventilatory criteria, which according to the Berlin definition [2], are classified as: 1. Mild: 201 mmHg < PaO_2_/FiO_2_ ≤ 300 mmHg, with PEEP/CPAP > 5 cm H_2_O; 2. Moderate: 101 mmHg < PaO_2_/FiO_2_ ≤ 200 mmHg, with PEEP/CPAP > 5 cm H_2_O; 3. Severe: PaO_2_/FiO_2_ ≤ 100 mmHg, with PEEP/CPAP > 5 cm H_2_O.

Nowadays it is not possible to anticipate the extent of the COVID-19 epidemic since the SARS-CoV-2 virus is highly contagious and spreads rapidly from person to person through cough or respiratory secretions, and therefore close contacts. Although it is true that with good health promotion, it can be prevented. An informed and updated population will be able to reduce the contagiousness of the virus. Respiratory drops of more than five microns are capable of being transmitted up to a distance of two meters, and from hands or fomites contaminated with these secretions followed by contact with the mucosa of the mouth, nose, or eyes. This new virus has a predilection for the respiratory tree, in which once it penetrates it generates an abnormal immune response of an inflammatory type with an increase in cytokines, which aggravates the patient and causes multiorgan damage. An emergency is “a sudden serious and dangerous event or situation which needs immediate action to deal with it” [3]. Considering this, those with COVID-19 pneumonia, who meet the Berlin criteria for ARDS, have an atypical form of the syndrome. The main features are the dissociation between his relatively well-preserved lung mechanics and the severity of the hypoxemia. Due to the consequent number of patients requiring intensive care management in an emergency situation, the health personnel of these services must be prepared to provide specific organ support treatments and consider that certain treatments may be necessary for a large number of patients. Clinical presentation, comorbidities, age, number of days on mechanical ventilation, and risk of complications are factors that influence a potentially favourable outcome [4,5].

Preparing for the COVID-19 pandemic requires a multidisciplinary, stakeholders approach, given that approximately 14% of people with COVID-19 develop a serious illness, which can include ARDS. The coordinated response of the emergency services through the WHO guidelines have facilitated coping with the virus in these two years. The demand for care has saturated the emergency and intensive care services, with material resources becoming scarce. For example, World Health Organization (WHO) guidelines suggest that COVID-19 patients with refractory hypoxemia, despite protective lung ventilation, be considered for extracorporeal life support (ECLS), which is a scarce resource that may require rationing in a pandemic situation [6]. COVID-19 infections are still growing in 52 countries. There have been at least 172,731,000 reported infections and 3,863,000 reported deaths caused by the new coronavirus to date. The region is currently reporting one million infections and has reported more than 52,173,000 cases since the pandemic began [7].

Emergency medical services personnel play a prominent role in the triage, transportation, and initial treatment of adult patients with respiratory distress. Compared to basic life support, prehospital care at the advanced life support level results in a decrease in mortality to 12.4% and a substantial improvement in symptom relief due to early therapeutic interventions [8]. Likewise, the care provided in the Intensive Care Units (ICU) is essential, since the complications associated with ARDS can be very serious [9,10].

Regardless of how the pandemic evolves, emergency and critical care personnel must be prepared and trained to apply early and optimal interventions. Extracorporeal organ support therapies can represent an important part of the response and healthcare professionals should be familiar with them. A call to action should be made to publicize the different techniques, each with specific criteria and modalities of prescription, delivery, and follow-up [11,12].

The main objective of this work was to analyze the management and evolution of ARDS in emergency situations in the last 5 years. In addition, it is essential to know the resources, promotion, and support in these situations, whether or not they are derived from the COVID-19 pandemic.

## 2. Materials and Methods

### 2.1. Search Strategy

A systematic review was carried out in the PubMed and Scopus databases. A systematic review was performed, in accordance with the PRISMA guidelines [13]. Using the descriptors Medical Subject Headings (MeSH), the search equation in both databases was: “Emergency health service AND acute respiratory distress syndrome”. In addition to the inclusion/exclusion criteria, in SCOPUS the search was filtered in abstract, title, and keywords. The search was conducted in December 2021. The study was registered (ID: 331782) in the PROSPERO database (International Prospective Register of Systematic Reviews).

The PICO (Patient, Intervention, Comparison, and Outcome) question was used. The population was patients with acute respiratory distress syndrome; the intervention was the initial management and during the ARDS crisis; the comparison was addressing the different resources, techniques, and support in emergency situations; and the outcome was the clinical benefits of each treatment. Therefore, the search question was: What are the therapeutic options in the management of patients with ARDS in emergency situations?

### 2.2. Study Selection

*Inclusion criteria*: quantitative primary studies on the care of patients with ARDS in an emergency setting were included, published in English and/or Spanish, and with restrictions between the years 2016–2021.

*Exclusion criteria:* Doctoral theses, articles without statistical information (prevalence, means, or standard deviation), duplicate studies, and articles whose objectives were not to investigate acute respiratory distress in emergency situations were also excluded. The selection of articles was carried out in 4 steps: reading the title and abstract, followed by reading the full text. Afterwards, a reverse search was carried out, from the references of the selected studies, and finally, a critical reading of the studies to assess their methodological quality.

### 2.3. Level of Evidence

The quality of the studies included in this review was assessed following the levels of evidence and grades of recommendation stipulated by the Oxford Center for Evidence- Based Medicine (OCEBM) [14]. The quality of the evidence assessment was performed independently and in duplicate by two reviewers. The OCEBM is a system that establishes a hierarchical order based on the methodological design of the research analysed. This allows to establish a level of scientific evidence and a degree of recommendation for each one.

### 2.4. Variables and Data Collection

To extract the data from each study, a data collection notebook was created that included the first author, year of publication, country of study, design, sample, main results, and the level of evidence/grade of recommendation.

## 3. Results

### 3.1. Characteristics of the Selected Studies

**Figure 1:**
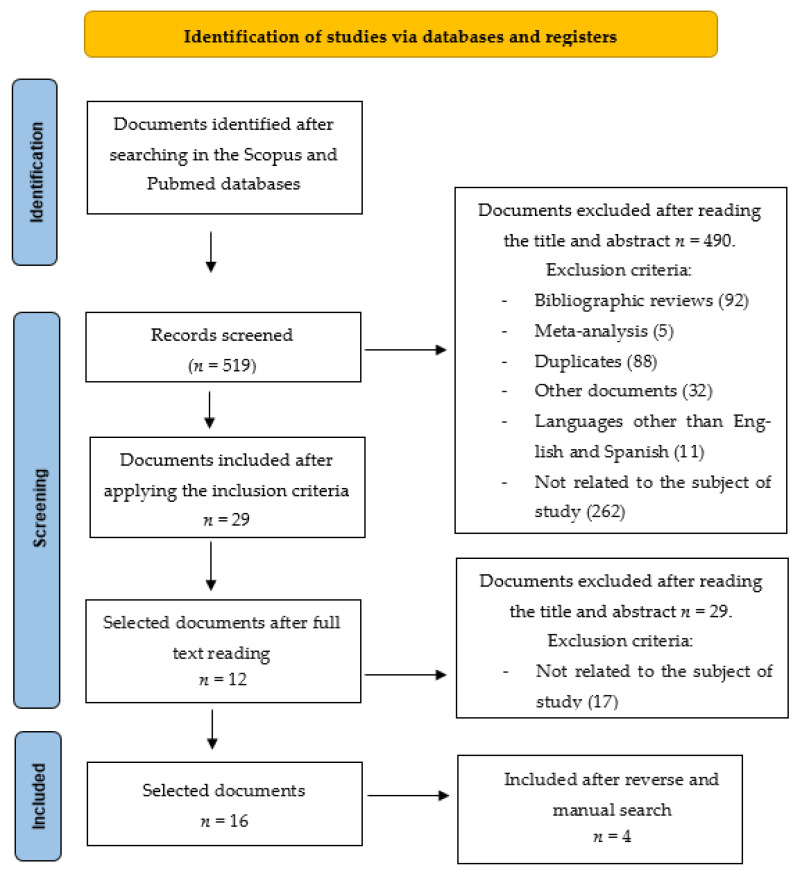
Selection process for reviewed articles.

A total of 519 articles were found in the databases. After reading the title and abstract, 402 studies were excluded. After discarding duplicates using the Mendeley filing system, 29 articles were finally examined. After full-time reading, 12 studies were selected. After conducting a reverse and manual search, 4 more articles were found, so 16 articles remained for study (Figure 1).

To extract the data from each study, a data collection notebook was created. All the studies involved prospective longitudinal cohorts, except for three of a retrospective type and one quasi-experimental study (Table 1).

### 3.2. Clinical Characteristics of ARDS

Several authors [23,28] highlight the importance of the characteristics and therapeutic management in the emergency and ICU service of patients diagnosed with ARDS. Field observations, along with recent publications, distinguish a peculiar presentation with severely hypoxemic patients who show no signs of clinical respiratory distress (“silent or happy” hypoxemia) while others exhibit a more conventional presentation of shortness of breath and much more serious hypoxemia. No differences were found in the pre-hospital management of the two patient phenotypes, but the length of stay in ICU and mechanical ventilation was greater in Phenotype 1, although mortality in ICU and hospital did not present differences in either of the two. The early evaluation and admission of these patients with silent hypoxemia to a high dependency unit are essential to avoid the progression of the disease towards a worse prognosis. Type 1 patients (silent or happy hypoxemia) could be mistakenly reassuring because they can present a rapid and brutal clinical deterioration that justifies their early admission to the ICU; in these studies they also report that therapy in the prone position would improve both the oxygenation rate and the prognosis of patients with Mechanical Ventilation but would also help patients with spontaneous ventilation significantly reduce the intubation rate and improve mortality.

Regarding the demographic data and characteristics of ARDS (age), a higher percentage of patients with high severity ARDS were included, and it was found that the overall mortality was 38% [19].

### 3.3. Ventilatory Support in ARDS

Nielsen [26] reports that in a pre-hospital setting, adherence to standard treatment and with pre-hospital CPAP was high in patients with ARDS. These patients experienced greater increases in SpO_2_ and reduced respiratory rate during prehospital transport compared to a cohort of patients treated with standard care alone. The study is useful in planning future trials and for systems intending to implement pre-hospital CPAP.

According to Fuller [20] Lung protective ventilation initiated in emergencies was positive for patients intubated in the ED who were at risk for ARDS and other ventilator-associated complications. Evidence shows that potentially harmful ventilation practices are common in the emergency department. This study demonstrated that the implementation of a mechanical ventilation protocol in the emergency department is feasible and was associated with improvements in the delivery of safe mechanical ventilation and in clinical outcomes. Innovation can improve the health of society only if it reaches the patient and has external validity.

### 3.4. Adjuvant Therapies in ARDS

Several studies allude to the importance of the use of ECMO for severe acute respiratory distress syndrome. This is used to decrease the intensity of mechanical ventilation or as salvage therapy in refractory ARDS. They show that older age and a longer delay from endotracheal intubation to the start of ECMO are associated with a worse outcome. The state of immunosuppression was also associated with poorer survival; in three of the studies, the scarce use of the prone position was striking, while the use of ECMO protective pulmonary ventilation was used in all study centers despite being a lot more expensive and complex. Therefore, early identification of this subgroup of ARDS patients before the onset of permanent lung damage and weakness will be crucial to improve outcomes. A simple ECMO prediction score that can be calculated using the commonly used ventilation and oxygenation variables available within the first 12 h of intubation for ARDS and the score obtained, provided highly effective discriminatory power to detect progression of ARDS at sufficient severity to consider ECMO [15,24,30]. Another study referred to the need for a fluid-conserving strategy to prevent fluid overload during the initial phase of ECMO in patients with severe ARDS [16].

Treatment of ARDS in Japan was characterized by a high rate of glucocorticoid use, which was positively associated with mortality. Two available pharmacological treatments were evaluated. Glucocorticoids were administered in more than half of the patients and their use was associated with poor research results. Furthermore, the glucocorticoid dose level was positively associated with mortality, regardless of severity. The efficacy of glucocorticoids for ARDS has long been a subject of debate and remains controversial even in guidelines.

Other authors confirm that the use of the prone position in both intubated and non-intubated patients with severe ARDS is associated with better survival in these patients [17,21,25]. Scaramuzzo et al. [29] also stated that after the first session in the prone position, oxygenation improves. This improves survival and decreases dependence on mechanical ventilation in the case of critically ill patients with COVID-19.

Fernández Tobar et al. [18] referred to the usefulness of the use of methadone for the control of “difficult sedation” refractory in treatment with other drugs during prolonged sedation of patients with ARDS, since prolonged use of sedatives and opiates is associated with the tolerance and dependence phenomena.

### 3.5. Optimization of Resources in Patients with ARDS in Emergency Situations

Two other articles [22,27] reported that the lack of resources sometimes forces a classification decision to be made on which patients are most likely to survive to the start of mechanical ventilation. This accurately reflects the realities of intensive care and emergency in a resource-limited setting. The development of ARDS and mortality at 28 days were lower than expected, and they did not find any suggestion of an association between pre-hospital vulnerability and these short-term outcomes, although it was strongly associated with mortality at one year in hospital-surviving patients.

### 3.6. Complications of Acute Respiratory Distress Syndrome in Children

Killien et al. [22], in relation to the complications of ARDS in children, reported that the duration of ventilation, ICU, and hospital stay were significantly longer among ARDS survivors. Tracheostomy placement occurred in 18.4% of ARDS survivors versus 2.1% of patients without ARDS. The development of ARDS in children after a traumatic injury is associated with an increased risk of morbidity and mortality, even after adjusting for injury severity and hemodynamic abnormalities. Patients with ARDS experienced higher rates of other hospital complications than patients without ARDS, such as pneumonia (20.6% vs. 2.3%), sepsis or bacteraemia (4.5% vs. 0.4%), other infections (4.5% vs. 1.1%), and cardiac arrest, while cardiac arrest was associated with an increased risk of death. Neither sepsis/bacteremia nor other infections were associated with a risk of death. All evaluated hospital complications were associated with an increased risk of post-discharge care. Among patients who survived to hospital discharge, the duration of mechanical ventilation, ICU duration, and hospital stay were significantly longer. Of the patients who survived ARDS, 56% required ongoing care after discharge. The most common rehabilitation among survivors was hospital type (29.7%), followed by care in long-term care centers (10.6%). The development of ARDS was significantly associated with morbidity and mortality for all causes, even after adjusting for major confounders.

## 4. Discussion

ARDS was already present as a serious health problem before the pandemic. In this manuscript, this syndrome’s presence in the last 5 years was analysed to see if the therapeutic approach is different if one suffers from COVID-19 disease or not. This helps prevent complications for patients and for exposed healthcare personnel. This first analysis offers controversial results, since the protocols currently continue to evolve just like the pandemic.

Some studies [31,32] specify that older age, male sex, and lower body mass index have a direct relationship with a higher mortality rate, in which the non-survivors are older and have low body weight with a higher risk of death. Mohammadi et al. [33] corroborate this fact and add chronic diseases such as high blood pressure and diabetes to age. In addition, these authors stated that there are no significant differences in the mortality rate between patients with early intubation and patients who are never intubated. Fayed et al. [34] agree on the latter, except in patients with a low SOFA score, who could benefit. Papoutsi et al. [35] even stated that the timing of intubation may have no effect on mortality and morbidity in critical COVID-19 patients. However, Laghlam et al. [36] discussed the importance of invasive and non-invasive mechanical ventilation in patients with ARDS. This allows to improve the respiratory parameters and reduce mortality [31]. Therefore, the early identification of ARDS patients before the onset of lung damage will be crucial to improving outcomes.

Regarding the use of the prone position, there are studies [36,37,38,39] that confirm our results since in this pandemic situation, the use of the prone position, both manual and with the use of the bed in the prone position, due to the decompensation of COVID-19 patients, is essential due to the lack of other types of more complex resources and expensive. However, other authors state that the prone position lengthens the MV time and stay in certain circumstances [39].

Several authors confirm that the ECMO system is necessary to allow the lungs to recover from their inhalation injuries, and to avoid harmful elevated ventilation strategies due to severe ARDS and improve respiratory parameters. The usefulness of ECMO is expanding with more research and technology, allowing ubiquity to become more and more apparent. It is important to have a basic understanding of ECMO, including the indications, contraindications, and evidence to support its use. ECMO can be life-saving in severe cases, when mechanical ventilation fails to maintain adequate oxygenation and it has been proven that it can reduce mortality [36,40,41,42].

In line with what was previously seen, Baltaji et al. [43] and Ronco et al. [44] support our results when they say that it is necessary to know the characteristics and therapeutic management in the emergency service of patients diagnosed with ARDS by COVID-19. The objective is to have an adequate approach in order of arrival, clinical presentation, comorbidities, age, and the time of mechanical ventilation because they are essential and influence the risk of complications for a potentially favourable outcome.

The lack of resources sometimes forces us to make a classification decision on patients who have a better chance of survival, and in this pandemic situation that we are experiencing, adequate prehospital care at the level of advanced life support in acute respiratory failure, results in a decrease in mortality and a substantial improvement in symptom relief due to early therapeutic interventions. Indeed, Kuljit et al. [45] confirm that the COVID-19 pandemic approach requires a multidisciplinary approach and a rational deployment of resources to ensure that scarce and expensive life-saving technology is available to as many patients as possible. For all the more reason, this policy is applied to those scarcer resources, as is the case of ECMO in the ICU [46].

Regarding the use of corticosteroids, there are studies that support our results. Specifically, Fernandes et al. [47] state that the use of corticosteroids did not lead to an improvement in patients suffering from COVID-19 and RECOVERY advises against their use for prevention. Dexamethasone is of value only in hospitalized patients requiring respiratory support. Therefore, it would be advisable to clarify the conditions of its use. However, Zhang et al. [48] stated that there is no association between the use of corticosteroids and ARDS mortality, and Rahman et al. [49] even commented that methylprednisolone can reduce mortality, length of hospital stay, and duration of mechanical ventilation.

As far as the pediatric population is concerned, therapeutic considerations vary substantially. ARDS in children after traumatic injury is associated with an increased risk of morbidity or mortality, even after adjusting for injury severity, and that the most common causes of ARDS in children are sepsis, aspiration, episodes of near-drowning, pneumonia, and trauma severity of hypoxemia defines the severity of ARDS. Naveda et al. [50] confirm that ARDS has different mortality rates between paediatric and adult patients, with different histological findings, and with different prognoses in ARDS triggered by similar causes between children and adults. These findings suggest that ARDS in paediatric patients could have a different course than in adult patients, thus alluding to the need for studies with risk factors specific to the paediatric population.

### 4.1. Future Practical Implications

Due to the haste with which it was necessary to deal with different pathologies due to COVID-19, among which those of a respiratory nature should be highlighted, there have been several international collaborative projects that have tested the effectiveness of different treatments. Among these projects, on the one hand, the Randomised, Embedded, Multifactorial, Adaptive Platform (REMAP) design stands out as an adaptive clinical trial, which, unlike conventional trials, avoids ambiguity of results; by accumulating data, it increases the probability that the patients within the trial are randomly assigned to treatments that are most likely to be beneficial; allows several questions to be tested simultaneously, etc. Among different press releases and publications derived from these types of designs, it is worth highlighting where it is stated that low-cost dexamethasone reduces death by up to a third in hospitalized patients with severe respiratory complications due to COVID-19. In fact, the above result is due to the other international collaborative project to be highlighted: the Randomised Evaluation of COVID-19 Therapy (RECOVERY) international clinical trial, which has aimed to identify treatments that may be beneficial for people hospitalized for COVID-19. These are just two examples of ongoing research work in this area [51].

For this reason, to prevent cases of ARDS and complications with a poor prognosis, early identification of patients at risk is very important. In addition, correct emergency management, diagnostic tests, and rational use of resources are essential. If there are risk factors and the evolution is rapid, ICU admission should be considered to minimize mobility and mortality [52].

### 4.2. Study Limitations

This study has several limitations, firstly, most of the studies were longitudinal, and therefore it is necessary to carry out randomized clinical trials in relation to ARDS in the initial management in the emergency for the approach of the COVID-19 patient.

## 5. Conclusions

It is essential to reduce variability and optimize pre-hospital care of the respiratory patient since this will reduce medical costs and improve the survival of these patients. For these reasons, proper health promotion will help prevent the syndrome in the population at risk. Overweight patients, chronic pathologies, and late management with oxygen therapy can develop ARDS.

The therapeutic options in atypical ARDS due to COVID-19 do not seem to vary substantially from conventional ARDS.

The management of ARDS in children varies with respect to adults and requires an exhaustive analysis to face it.

Interventions for ARDS offer controversial results and it is not clear whether their use in one way or another improves prognosis. The different cases can significantly influence the outcome, so more studies must be carried out to reach a consensus and disseminate them through international initiatives such as REMAP and RECOVERY.

## Figures and Tables

**Table 1:** Characteristics of the selected studies.

Author/Year (Country)	Design	Sample	Aims	Support and Technique	Results	Level of Evidence/Grade of Recommendation
Bohman et al. [15], 2016. (EEUU)	Observational prospective	767 patients	Identify and classify patients with newly diagnosed acute respiratory distress syndrome (ARDS) who may progress to severe ARDS.	ECMO	Data-Driven Early Prediction ECMO Eligibility for Severe ARDS Score commonly uses variables from ARDS patients within 12 h of intubation and could be used to identify patients who may merit early transfer to a center ECMO-trained physician.	2c/B
Chiu et al. [16], 2021. (China)	Observational prospective	152 patients	To investigate the effect of cumulative fluid balance during the early phase of ECMO on clinical outcomes and hospital mortality in patients with severe ARDS		There was a stepwise increase in hospital mortality corresponding to an increase in CFB quartile, with significant between-group differences in terms of 28-, 60-, and 90-day hospital mortality (all *p* < 0.05). Patients in lower CFB quartiles presented more ECMO-free days by day 28; however, the effect was not significant. We observed significantly higher 28- and 60-day ventilator-free days in lower CFB quartiles (*p* = 0.002 and 0.001, respectively). We also observed significantly lower overall 90-day survival rates in quartile 4 (overall comparison, *p* = 0.001, log-rank test), as follows: quartile 1 (63.2%), quartile 2 (55.3%), quartile 3 (50%), and quartile 4 (31.6%).	2c/B
Ehrmann et al. [17], 2021. (Canada, EEUU, France, Ireland, Mexico, Spain)	Randomized Controlled Trial	1126 patients	To evaluate the efficacy of awake prone positioning to prevent intubation or death in patients with severe COVID-19 in a large-scale randomised trial.	Prone positioning	Treatment failure occurred in 223 (40%) of 564 patients assigned to awake prone positioning and in 257 (46%) of 557 patients assigned to standard care (relative risk 0·86 [95% CI 0·75–0·98]). The hazard ratio for intubation was 0·75 (0·62–0·91), and the HR for mortality was 0·87 (0·68–1·11) with awake prone positioning compared with standard care within 28 days of enrolment. The incidence of prespecified adverse events was low and similar in both groups.	1b/A
Fernández Tobar et al. [18], 2016. (Spain)	Observational retrospective	13 patients	To describe the experience with the use of methadone for the control of difficult sedation (SAD), in patients ventilated for secondary ARDS COVID-19 in whom it has been failed with the use of the usual drugs.	Use of methadone	85% of the patients improved the quality of sedation-analgesia, achieving values -2 and 0 on the RASS scale (Richmond Agitation-Sedation Scale), pain control, with a visual numerical scale <4 and the dose could be reduced of drugs used for adaptation to MV. Methadone is a drug to be considered in the management of SAD pictures secondary to the administration of high and prolonged doses of sedative drugs and opiates during MV in patients with ARDS.	2c/B
Fujishima et al. [19], 2020. (Japan)	Observational prospective	166 patients	To examine therapeutic strategies for ARDS.	Mechanic ventilation	The proportion of patients with PaO_2_/FIO_2_ ≤ 100, patients under positive pressure invasive ventilation, and in-hospital mortality was 39.2%, 92.2%, and 38.0% for American—European Consensus Conference acute lung injury criteria. As well, 38.9%, 96.8%, and 37.6% for patients with Berlin definition ARDS, respectively.	2c/B
Fuller et al. [20], 2018. (EEUU)	Quasi-experimental	229 patients	To assess the impact of mechanical ventilation in an emergency department (ED). Protocol on clinical outcomes and adherence to lung protective ventilation in patients with ARDS.	Mechanical Ventilation Protocol (1) protective tidal volume of the lungs; (2) appropriate setting of positive pressure at the end of expiration (PEEP); (3) weaning from oxygen; and (4) elevation of the head of the bed)	The mechanical ventilation protocol was associated with a reduction in mortality from 54.8% to 39.5% (OR 0.38, 95% CI 0.17–0.83, *p* = 0.02) and a 3.9-day increase in ventilator-free days, *p* = 0.01.	2c/B
Guervilly et al. [21], 2019. (EEUU)	Observational retrospective	168 patients	To compare the results of patients with severe ARDS under ECMO according to the use of Prone Position or lack of it during their execution of ECMO.	Prone and ulnar position and ECMO	Patients in the prone ECMO group were more likely to be weaned from ECMO. Consequently, the 30-day, 60-day, and 90-day survival rates were significantly higher.	2c/B
Killien et al [22], 2019. (Canada and EEUU)	Observational prospective	146,058 patients	To assess morbidity and mortality in children with ARDS.	Hospital mortality and the need for post-discharge care	Mortality was 20.0% among patients with ARDS versus 4.3% among patients without ARDS. Post-discharge care was required in an additional 44.8% of patients with ARDS versus 16.0% of patients without ARDS (aRR 3.59, 2.87–4.49).	2c/B
Le Borgne et al. [23], 2020. (France)	Observational retrospective	103 patients	To describe the characteristics and therapeutic management of the mobile emergency service of patients with vital distress due to COVID-19, their hospital care pathway and their in-hospital evolution.	Mechanic ventilation	Serious SARS-CoV-2 infections have revealed two different clinical presentations. The first phenotype (“happy” hypoxemia) should be managed similarly to the second phenotype (hypoxemia with clinical acute respiratory failure) which includes early admission to the ICU or close supervision in a high dependency unit for appropriate life support. The clinical phenotypes appear to be highly differentiable in the pre-hospital setting, but no differences were found in terms of mortality; therefore, identical management is recommended in the initial phase.	2c/B
Li et al. [24], 2021. (Thailand)	Observational retrospective	31 patients	To investigate the timing of ECMO initiation in critically ill patients with COVID-19.	ECMO	The 60-day mortality rate after ECMO was 71% and the weaning rate from ECMO was 26%. The early initiation of ECMO was associated with a decrease in mortality at 60 days after ECMO (50 vs. 88%, *p* = 0.044) and an increase in the weaning rate of ECMO (50 vs. 6%, *p* = 0.011).	2c/B
Loureiro-Amigo et al. [25], 2021. (Spain)	Observational retrospective	163 patients	To assess the impact on hospital mortality of the prone position in spontaneously breathing patients with COVID-19 and severe ARDS.	Prone positioning	Patients treated with the prone position had lower mortality (62.1% vs. 43.3%, *p* = 0.0229), with an estimated OR of 0.47 (95% CI: 0.24 to 0.89). The use of the prone position showed a protective effect on mortality (OR 0.42, 95% CI 0.18 to 0.98).	2c/B
Nielsen et al. [26], 2016. (Denmark)	Observational prospective	171 patients	To assess adherence to treatment and the efficacy of CPAP as an addition to standard care.	Continuous positive airway pressure CPAP	Patients with CPAP had a greater increase in SpO2 than patients without CPAP (87 to 96% versus 92 to 96%, *p* < 0.01) and a greater decrease in respiratory rate (32 to 25 versus 28 to 24 breaths/min, *p* < 0.01.	2c/B
Osei-Ampofo [27] et al, 2018. (Ghana)	Observational prospective	82 patients	To assess the incidence of respiratory failure requiring mechanical ventilation and the presence and Outcomes of ARDS.	Mechanic ventilation	In this study, intubation and mechanical ventilation were performed in 9% of critically ill patients. While only 2.4% of the intubated patients met the criteria for ARDS.	2c/B
Piva et al. [28], 2020. (Italy)	Observational prospective	44 patients	To analyse the experience in caring for COVID-19 patients.	Use of invasive, non-invasive ventilation, and adjuvant therapies for the treatment of COVID-19	Non-invasive ventilation was performed in 39% of the patients during part or all of their stay in the ICU without infection of the patient. 97% of the patients required FiO_2_ ≥50% upon admission to the ICU, with a mean of 80%, although the patients were able to oxygenate with adequate SaO2 values. Severe ARDS, with PaO_2_/FiO_2_ <150 mmHg, was present in 64% of the patients. 39% of the patients were managed with non-invasive positive pressure ventilation for part or all of their stay in the ICU. Patients who received invasive mechanical ventilation were ventilated with low tidal volume ventilation.	2c/B
Scaramuzzo et al. [29], 2021. (Italy)	Observational prospective	470 patients	To analyze whether the variation in oxygenation after the first prone positioning session, compared to the pre- prone positioning state, could be associated with ventilation-free days (VFD) in the ICU, mortality in the ICU and the probability of release of mechanical ventilation evaluated at 28 days after admission to the ICU.	Prone positioning	The median PaO_2_/FiO_2_ variation after the first PP cycle was 49 [19–100%] and no differences were found in demographics, comorbidities, ventilatory treatment and PaO_2_/FiO_2_ before prone positioning between responders (96/191) and non-responders (95/191). Moreover, oxygenation response after the first positioning was independently associated to liberation from mechanical ventilation at 28 days and was increasingly higher being higher the oxygenation response to PP	2c/B
Schimdt et al. [30], 2018. (France)	Prospective observational cohort	83 patients	To describe the ventilatory management, characteristics and outcome of patients treated with ECMO with ARDS.	ECMO	ECMO must be considered for patients who develop refractory respiratory failure.	2c/B

Remark = CPAP: continuous positive airway pressure; ECMO: extracorporeal membrane oxygenation; ARDS = Acute respiratory distress; VM = Mechanic ventilation.

## Data Availability

The data presented in this study is available by contacting the corresponding author.
